# *De novo* sensitization during subcutaneous allergen specific immunotherapy - an analysis of 51 cases of SCIT and 33 symptomatically treated controls

**DOI:** 10.1038/s41598-020-63087-4

**Published:** 2020-04-08

**Authors:** Donata Gellrich, Katharina Eder, Catalina Högerle, Sven Becker, Martin Canis, Moritz Gröger

**Affiliations:** 1 Department of Otorhinolaryngology, Head and Neck Surgery, Ludwig-Maximilians-University Munich, Munich, Germany; 2 Department of Otorhinolaryngology, Head and Neck Surgery, University Medical Center of Eberhard Karls University Tuebingen, Tuebingen, Germany

**Keywords:** Immunization, Outcomes research, Respiratory signs and symptoms

## Abstract

Since the beneficial implementation of allergen specific subcutaneous immunotherapy (SCIT), there are only a few studies on the risk of SCIT-induced neosensitizations. In 51 patients, we retrospectively analyzed sIgE and sIgG patterns by a multiplex ELISA as well as demographic and clinical features before and after SCIT. 33 allergic patients, who only received symptomatic treatment, served as controls. In 12 of 51 SCIT-treated patients (24%), we found new sIgE against allergen components of the allergen source treated by SCIT; eight of them were adults. Among controls, no adult patient showed neosensitization to components of the primarily affected allergen source. Only two children of the control group were affected by neosensitization, which was limited to major allergen components and rarely accompanied by sIgG. In the SCIT-treated group, neosensitization affected major and minor allergen components, and was accompanied by a strong induction of sIgG against major components. A clear clinical predictor of neosensitization during SCIT was not found. Comparing symptom scores, patients seem to profit more from SCIT, if neosensitization remained absent. Patients undergoing SCIT might carry an enhanced risk of neosensitization towards formerly unrecognized allergen components. According to anamnestic data, these neosensitizations might be of clinical relevance - supporting attempts towards personalized recombinant vaccines.

## Introduction

Since the implementation of allergen specific immunotherapy (SIT) in clinical practice, many studies have been conducted to analyze the immunological changes during SIT^1^. Many publications focus on the antibody responses induced by SIT in general and the antibody shift from IgE to IgG subclasses in particular^2^.

However, there are only a few studies on the potential induction of new sensitizations by SIT. The exposure to allergens, especially if combined with adjuvants, might lead to new sensitization to formerly unrecognized allergen epitopes^3,4^. With regard to venom SIT, the neosensitization to minor allergens has been described^5^. Concerning other important allergen sources, conflicting results are found: Regarding house dust mite (HDM) and grass pollen, the percentage of *de novo* sensitizations toward minor allergens differ from 0%^6–10^ to 8–12%^11–13^.

One major influencing variable might be the observation period: Studies with short-term observation after one course of SIT or less tend to exclude neosensitization^7–10^, possibly by missing middle- or long-term immunological changes occurring only after longer period of repetitive exposure to the allergen. However, all studies reporting *de novo* sensitization induced by SIT had a longer observation period^12,13^ except for the publication by Baron-Bodo *et al*. in 2013^11^ detecting neosensitization in 10% of patients treated by grass pollen sublingual immunotherapy (SLIT) for four months only. With regard to *de novo* sensitization induced by birch pollen SIT, all three published studies report on cases of neosensitization^14–16^.

Another factor, which might affect the detection rate of *de novo* sensitization during SIT, is the diversity of allergen components integrated in the measurements of sIgE. Most studies on neosensitization by SIT measured sIgE only against a few allergen components carrying the risk of missing newly induced sIgE against non-tested allergens.

As time and diversity of sIgE serology appear to be of high relevance in the SIT-induced neosensitization, we initiated the present study: In 51 allergic patients, the sensitization pattern was analyzed before and after the end of their subcutaneous specific immunotherapy (SCIT) by means of a multiplex microarray test, which enabled us to detect sensitizations to allergen extracts and various different molecular allergen components, a unique variety of allergens, which is commercially not available by any other diagnostic system. All patients had maintenance treatment for at least 2.8 years prior to the second examination in order not to miss immunological changes, which might occur only after many repetitive applications. As the clinical relevance of *de novo* sensitization is not analyzed in most studies and, therefore, remains unclear, we also investigated the clinical course of all patients. In contrast to all other studies on the potential induction of neosensitization in the course of SCIT, we included a control group comprised of 33 allergic patients, who received symptomatic treatment only.

## Patients And Methods

### Study population and design

The allergy database of our ENT department was scanned for patients with proven allergy, who had been treated by subcutaneous specific immunotherapy. In 51 cases, frozen serum probes from blood samples before the start and after the end of SCIT were available for retrospective total IgE and allergen-specific IgE analysis. These 51 patients were matched to 33 control patients, who were not treated by SCIT, but of whom two blood samples had been taken and frozen in the course of several years. In the control group, the mean time period between both blood samples was 5.4 years compared to 3.8 years in the SCIT-treated group. In all 84 cases, total IgE and allergen-specific IgE was analyzed in serum at both time points. Additionally, allergen-specific IgG was determined at both time points, except for three control patients of whom one serum sample’s volume sufficed for allergen-specific IgE analysis only. Further, the medical history was registered in all patients, partially by a standardized clinical history questionnaire comprising complaints of the upper and lower airways, oral and gastro-intestinal allergy symptoms as well as demographic data. In order to assess the severity of allergic disease, the clinical symptoms were assessed by a standardized questionnaire asking the patients to rank the severity of different rhinitis-related symptoms on a scale between 0 (no symptom) to 3 (massive symptoms).

In addition to sIgE serology, all 84 patients underwent skin prick test (SPT; ALK-Abelló, Wedel, Germany) at first consultation. Prior to SCIT, all 20 HDM-allergic patients underwent a positive allergen-specific provocation tests with intranasal challenge test solution for *D. pteronyssinus* (LETI Pharma GmbH, Ismaning, Germany). In the 64 patients with allergy against birch or grass, an allergen-specific provocation test was only carried out in unclear cases, for example in case of sensitization to several allergen sources with overlapping pollen seasons (i.e. birch and ash).

### Ethical approval and informed consent

The study was approved by the local ethics committee (Ethikkommission of the Ludwig-Maximilians-University, Germany, project number 18–448 UE) and the local data protection commissioner and was performed in accordance with all relevant guidelines and regulations. All patients provided written informed consent.

### Allergen-specific immunotherapy

18 patients received subcutaneous immunotherapy with *Fagales*; 12 patients were treated by a native allergen preparation and 6 patients by an allergoid. 19 patients underwent subcutaneous immunotherapy with *Poaceae* of whom 14 patients were treated by a native allergen preparation and 5 by an allergoid. 14 patients received subcutaneous HDM immunotherapy; 8 patients were treated by a native allergen preparation and 6 patients by an allergoid.

On average, the SCIT was carried out for 3.8 years. All patients had maintenance treatment for at least 2.8 years prior to the second examination.

The 33 control patients, of whom 12 were allergic to birch, 15 to grass und 6 to HDM, did not receive SCIT.

### ***In vitro*multiplex measurements (ALEX)**

Total IgE and sIgE reactivity to purified natural allergen extract and allergen components were measured using ALEX^®^, a multiplex ELISA test (ALEX Allergen Explorer, MADx MacroArray Diagnostics, Vienna, Austria) allowing measurement of total IgE and specific IgE (sIgE) against >150 allergen extracts and >100 molecular allergen components. With respect to *Fagales*, sIgE against allergen extract, Bet v 1, Bet v 2, and Bet v 6 were determined; relating to *Poaceae*, sensitivity towards allergen extract, Phl p 1, Phl p 2, Phl p 5b, Phl p 6, Phl p 7, and Phl p 12 was assessed; regarding HDM, sIgE against Der p 1, Der p 2, Der p 5, Der p 7, Der p 10, Der p 11, and Der p 23 were measured. In the ALEX assay protocol, a cross-reactive carbohydrate-determinants (CCDs) inhibitor is integrated during serum incubation to avoid misinterpretation of positive results due to cross-reactivity^17,18^. Results were reported as concentrations (kU/l). Concentrations ≥ 0.3 kU/l were considered as positive. As there is no clear cut-off between background noise and weak positive results in the ALEX^®^ multiplex test, an increase in sIgE titer was only considered as neosensitization when starting from a serum titre of 0.0 kU/l prior to therapy and ending at ≥ 0.3 kU/l after treatment.

In the ALEX^®^ multiplex ELISA test, sIgE and sIgG can competitively bind to the test allergen; therefore, sIgE values could be false-negative due to strongly increased sIgG levels. In order to exclude this bias, all serum probes also underwent sIgG measurements by a multiplex ELISA test by the same manufacturer (ALEX Allergen Explorer, MADx MacroArray Diagnostics, Vienna, Austria). According to the manufacturer, an increase in the sIgG-titer by more than 1,000 units can be regarded as a significant increase. In three patients of the control groups, the sIgG determination was not possible at both time points, as the second serum probe contained sufficient volume for the sIgE measurement only.

In six controls, a high sIgG-increase was registered in (in sum) ten single allergen component despite negative sIgE; consequently, the negative sIgE-titer could theoretically be false negative. As, in the FEIA method (UniCAP-FEIA, Thermo Fisher Scientific, Freiburg, Germany), the content of test allergen is high enough to exclude competitive binding of sIgG and sIgE, we re-tested sIgE using the FEIA method. In five of concerned ten single components, sufficient serum volume remained for a re-testing using the FEIA method with a commercially available test kit (UniCAP-FEIA, Thermo Fisher Scientific, Freiburg, Germany). In all re-tested five single components, the negative sIgE-titer was confirmed.

### Statistical analysis

Statistical analysis was performed with SigmaStat (Jandel Corp., San Rafael, CA, USA). For descriptive statistics we used mean values with standard deviation (SD). Concerning the total serum IgE, the median was calculated due to a few very high outlier values (>3.000 kU/l).

As all of the data failed normality testing, non-parametric tests have been used: The Mann-Whitney-U-test was carried out to compare the symptom scores between two different cohorts at the same time-point. The Wilcoxon-Test was used to compare the symptom score at different time-points among one study group.

To compare the distribution of categorical data among different groups, the chi-squared-test was carried out.

A p-value ≤ 0.05 was considered as significant.

## Results

### Characterization of the study population

The number of patients was differing in both cohorts, as the patients’ selection was based on retrospective data in the ENT database, and this database did not offer a higher number than 33 control cases to be matched. The latency between both blood samples was discrepant in both groups due to inhomogeneous time-points of the second blood withdrawal: A minimum time of 2.8 years between both blood samples was kept as necessary inclusion criterion in both groups in order not to miss long-term effects. In SCIT-treated patients, the second time-point of blood withdrawal was given by the end of SCIT after 3.8 years of treatment in average. However, in the control group, the mean latency between both blood samples was longer (5.4 years), which was tolerated assuming that a longer observation period might enhance the probability of newly acquired sensitizations.

Demographic and clinical data of both study cohorts are given in detail in Tables 1–3, separated according to the affected allergen source: *Fagales* (Table 1), *Poaceae* (Table 2) and HDM (Table 3). Concerning most parameters, the SCIT-treated group and the control group were comparable. However, concerning HDM-allergic patients, some discrepancies were detectable between the 14 SCIT-treated patients and the small control cohort, which was comprised of only six patients. Regardless of the treated allergen source, patients receiving SCIT showed a clear male predominance compared to symptomatically treated patients.

**Table 1:** Demographic, clinical and serological data of the SCIT-treated study group and the control cohort - with allergy to *Fagales*.

Characteristics	Study group treated by SCIT with *Fagales* (n = 18)	Control group without SCIT with *Fagales* (n = 12)
Gender		
Male	13 (72%)	4 (33%)
Female	5 (28%)	8 (67%)
Age (yrs)	36 (range: 12–66)	39 (range: 16–64)
Children (<12 years old)	0 (0%)	0 (0%)
Number of allergen sources sensitized to	3.3	2.9
Most common co-sensitization, against		
Poaceae	13 (72%)	8 (67%)
Oleaceae	6 (33%)	3 (25%)
House dust mite	4 (22%)	4 (33%)
Cat	4 (22%)	2 (17%)
OAS related to various allergen sources	8 (44%)	5 (42%)
Asthmatic complaints	5 (28%)	4 (33%)
Atopic dermatitis	3 (17%)	3 (25%)
Total serum IgE [kU/l]	124 (range: 6–2210)	157 (range: 8–719)
Prevalence of sIgE, prior to therapy, against		
Bet v 1	18 (100%)	12 (100%)
Bet v 2	3 (17%)	2 (17%)
Bet v 6	0 (0%)	0 (0%)

Age is given as mean and range, number of allergen sources sensitized to is given as mean and total serum IgE is given as median and range. All other values are number of patients total and percent of each evaluated subgroup.

Many parameters were comparable in the SCIT-treated study group and the control cohort.

**Table 2:** Demographic, clinical and serological data of the SCIT-treated study group and the control cohort - with allergy to *Poaceae*.

Characteristics	Study group treated by SCIT with *Poaceae* (n = 19)	Control group without SCIT with *Poaceae* (n = 15)
Gender		
Male	15 (79%)	8 (53%)
Female	4 (21%)	7 (47%)
Age (yrs)	29 (range: 7–66)	31 (range: 7–57)
Children (<12 years old)	2 (11%)	1 (7%)
Number of allergen sources sensitized to	3.7	2.9
Most common co-sensitization against		
Fagales	14 (74%)	8 (53%)
House dust mite	9 (47%)	6 (40%)
Cat	5 (26%)	4 (27%)
Oleaceae	4 (21%)	3 (20%)
OAS related to various allergen sources	7 (37%)	6 (40%)
Asthmatic complaints	5 (26%)	5 (33%)
Atopic dermatitis	2 (11%)	2 (13%)
Total serum IgE [kU/l]	196 (range: 13–1978)	238 (range: 35–726)
Prevalence of sIgE, prior to therapy, against		
Phl p 1	17 (89%)	14 (93%)
Phl p 2	8 (42%)	3 (20%)
Phl p 5b	10 (53%)	9 (60%)
Phl p 6	7 (37%)	7 (47%)
Phl p 7	1 (5%)	0 (0%)
Phl p 12	1 (5%)	2 (13%)

Age is given as mean and range, number of allergen sources sensitized to is given as mean and total serum IgE is given as median and range. All other values are number of patients total and percent of each evaluated subgroup.

Many parameters were comparable in the SCIT-treated study group and the control cohort.

**Table 3:** Demographic, clinical and serological data of the SCIT-treated study group and the control cohort - with allergy to HDM.

Characteristics	Study group treated by SCIT with HDM (n = 14)	Control group without SCIT with HDM (n = 6)
Gender		
Male	10 (71%)	5 (83%)
Female	4 (29%)	1 (17%)
Age (yrs)	23 (range: 6–44)	14 (range: 6–24)
Children <12 years old	4 (29%)	2 (33%)
Number of allergen sources sensitized to	2.8	2.8
Most common co-sensitization against		
Poaceae	8 (57%)	3 (50%)
Fagales	7 (50%)	1 (17%)
Cat	3 (21%)	1 (17%)
Oleaceae	2 (14%)	1 (17%)
OAS related to various allergen sources	3 (21%)	1 (17%)
Asthmatic complaints	4 (29%)	0 (0%)
Atopic dermatitis	1 (7%)	0 (0%)
Total serum IgE [kU/l]	222 (range: 8–2103)	217 (range: 17–470)
Prevalence of sIgE, prior to therapy, against		
Der p 1	12 (86%)	4 (67%)
Der p 2	12 (86%)	5 (83%)
Der p 23	10 (71%)	5 (83%)
Der p 5	5 (36%)	1 (17%)
Der p 7	4 (29%)	2 (33%)
Der p 10	2 (14%)	0 (0%)
Der p 11	2 (14%)	0 (0%)

Age is given as mean and range, number of allergen sources sensitized to is given as mean and and total serum IgE is given as median and range. All other values are number of patients total and percent of each evaluated subgroup.

Many parameters were comparable in the SCIT-treated study group and the control cohort.

### sIgE-patterns prior to therapy

As shown in Tables 1–3, SCIT-treated patients and controls exhibited a similar prevalence of sIgE against allergen components of the affected allergen source: Among patients allergic to *Fagales*, the prevalence of sIgE against birch allergen components was equal in the SCIT group and the control cohort at the initial measurement. Among *Poaceae-*allergic patients, the prevalence of sIgE against grass allergen components slightly differed between the SCIT group and the control cohort. Similarly, slight differences were found at the initial measurement in the prevalence of sIgE against HDM-allergen components in the HDM-SCIT group and the HDM allergic control cohort.

### Changes in sIgE-patterns throughout therapy

Among the 51 SCIT-treated cases, 12 were affected by neosensitization to allergen components of the treated allergen source (*Fagales* or *Poaceae* or HDM). This rate of neosensitization of 24% is significantly higher than the rate of neosensitization of 6% among the 33 control cases (p = 0.036). This statistical significance even increases (to p = 0.013) when excluding children from both cohorts, as, in the SCIT-group, eight of the 12 affected patients were adults, whereas, in the control group, no adult had experienced neosensitization.

**Figure 1:**
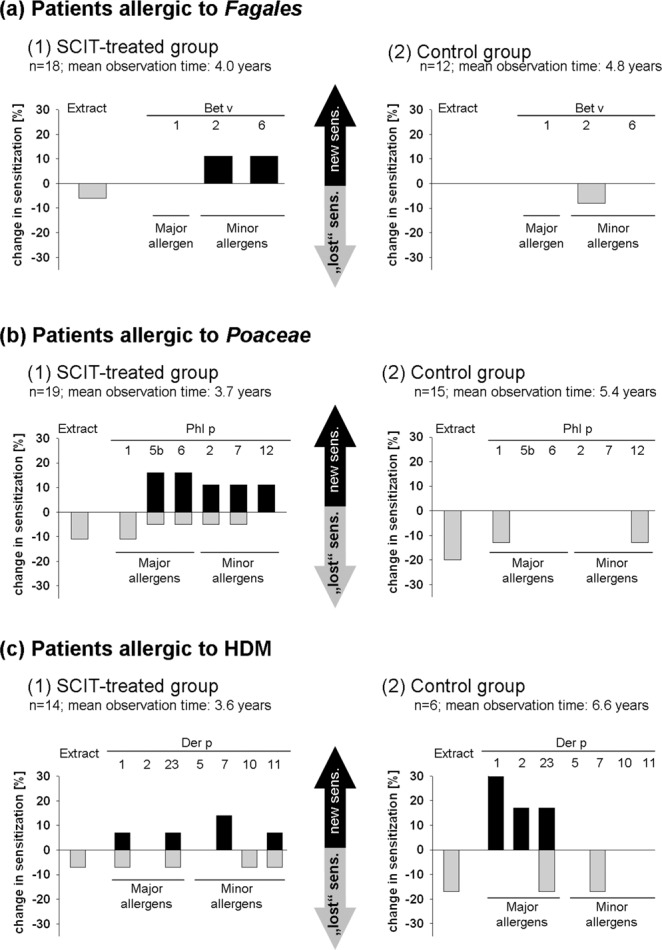
Changes in sIgE to extract and components throughout therapy in SCIT-treated patients (part 1) and control patients (part 2). Rate of *de novo* sensitizations (black bars) and rate of sensitizations reduced below the cutoff value (grey bars) throughout observation time: (**a**) in 30 patients allergic to *Fagales* (18 patients treated by SCIT with *Fagales* (1) and 12 *Fagales*-allergic control patients (2), given for birch extract and all measured birch allergen components), (**b**) in 34 patients allergic to *Poaceae* (19 patients treated by SCIT with *Poaceae* (part 1) and 15 *Poaceae*-allergic control patients (part 2), given for timothy grass extract and all measured timothy grass components), (**c**) in 20 HDM-allergic patients (14 patients treated by SCIT with HDM (part 1) and 6 HDM-allergic control patients (part 2), given for HDM extract and all measured HDM components). Sensitization lost below the cutoff value, was found in all study cohorts. Neosensitization to the treated allergen components was seen in all three SCIT-groups, but only in one control cohort (allergic to HDM, Fig. 1(c), part (2)). Further, the number and variety of allergen components affected by neosensitization differs in the study cohorts.

Figure 1, part (a) shows the rate of new sensitization against the birch allergen extract and against all measured birch allergen components as well as the rate of lost sensitization below the cutoff value in the *Fagales*-SCIT-treated group (part 1) as well as in the *Fagales*-allergic control group (part 2). The same analysis has been performed in patients allergic to *Poaceae* and HDM and is shown in Fig. 1, part (b) and Fig. 1, part (c), respectively.

In the SCIT-treated group, regardless of the treated allergen source (*Fagales* or *Poaceae* or HDM), neosensitizations applied to major and minor allergen components. The number of allergen components affected by *de novo* sensitization varied inter-individually among the patients: During SCIT with *Poaceae*, four patients developed new sIgE against one grass allergen component, two patients against two grass allergen components and one patient against four grass allergen components. After SCIT with *Fagales*, two patients exhibited new sIgE against one birch allergen component and one patient against two birch allergen components. During SCIT with HDM, one patient showed new sIgE against one HDM allergen component and another patient against three HDM allergen components. In sum, the variety of neosensitization towards eleven SCIT-related allergen components occured in 12 patients out of 51 (24%). The remaining cases did not show new sensitizations after treatment. In the control cohort, neosensitization was found in two children allergic to HDM and limited to the major allergens Der p 1, 2 and 23. No control patients experienced neosensitization to any minor allergen component. Further, when comparing the study group and the control cohort, it becomes obvious that the rate of sensitizations reduced to below the cutoff value throughout time is slightly higher in the control group than in the SCIT-treated group.

### Induction of sIgG throughout therapy

**Figure 2:**
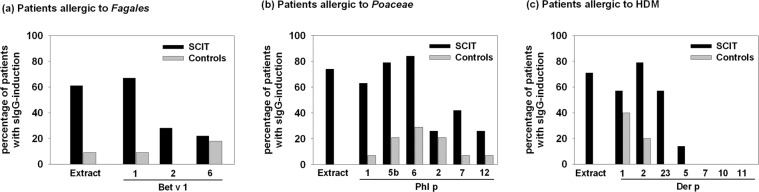
sIgG-induction in SCIT-treated patients vs. controls with allergy to (**a**) *Fagales*, (**b**) *Poaceae* and (**c**) HDM. In all groups (**a-c**), the induction of sIgG was more frequently observed in SCIT-treated patients (black bars) than in symptomatically treated control patients (grey bars).

In the total study population, sIgG increases were more commonly found in patients after SCIT compared to symptomatically treated patients, as shown in Fig. 2. During SCIT, the induction of sIgE to major allergen components was predominantly accompanied by induction of corresponding sIgG. However, in the two control patients with neosensitization a simultaneous induction of sIgE and corresponding sIgG was rarely seen.

Both types of allergen preparation administered during SCIT (native vs. allergoid) induced sIgG to major allergens. However, concerning the induction of sIgG to minor allergens, discrepancies were found: sIgG to Bet v 6, Phl p 2 and Phl p 12 were only registered after the administration of native allergen preparations, and none of the administered SCIT preparations – neither native nor allergoid - resulted in sIgG to the minor allergens Der p 7, 10 and 11.

### Clinical characterization of patients with neosensitization during SCIT

In order to identify a clinical predictor for an increased risk of neosensitization to allergen components contained in the treatment solution we analyzed demographic and clinical data of all 12 patients with neosensitization and compared the data with the characteristics of the remaining 39 patients without new therapy-related sensitizations despite SCIT.

**Figure 3:**
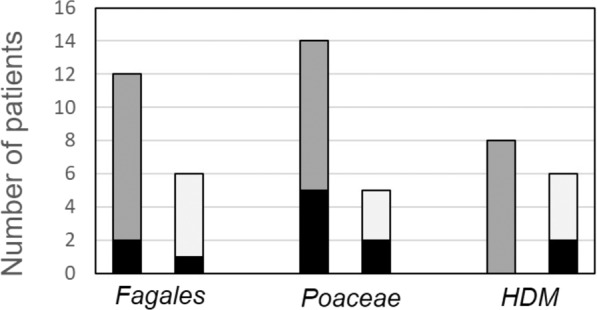
The rate of sIgE-neosensitization to treatment-related allergens according to the administered type of allergen preparation. In SCIT with *Fagales* and *Poaceae*, both allergen preparations, native (grey bars) as well as allergoids (white bars), led to neosensitization (black bars). In HDM-SCIT, only allergoid-treated patients (white bars) experienced sIgE-neosensitization to treatment-related allergens (black bars).

In the SCIT-group, the age in the beginning of SCIT does not seem to be a key factor of neosensitization as patients of all age groups were affected (range: 6–48 years). Further, the grade of sensitization as well as the correlating total serum IgE level does not allow a clear prediction: Among the 12 SCIT-treated patients with therapy-related neosensitization, patients with all grades of sensitizations were found from monosensitization to oligo- and polysensitization – correlating with total IgE levels from 6 kU/l to 2210 kU/l. Further, the type of administered allergen preparation does not seem to be a risk factor: After therapy with an allergoid, neosensitization to components of the treated allergen source was found in 29% (5 of 17 cases), after therapy with a native allergen preparation in 21% (7 of 34 patients), as given in Fig. 3.

### Clinical symptoms before vs. after therapy

**Figure 4:**
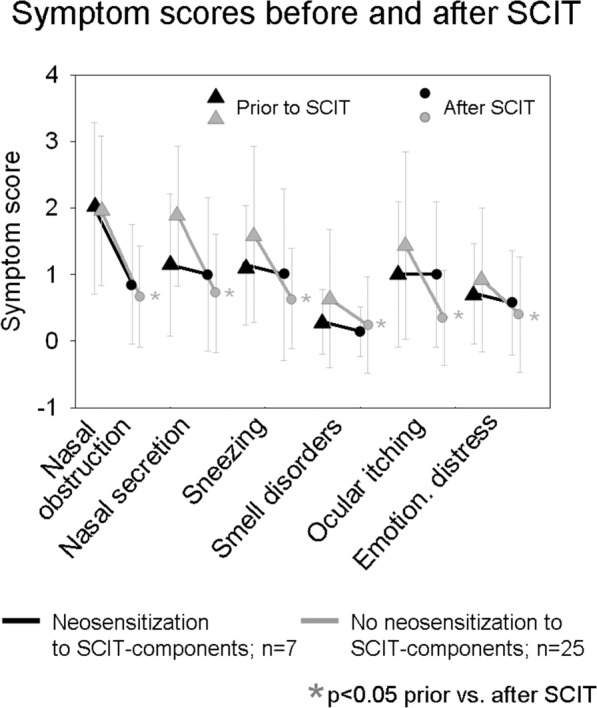
Change of symptom scores in the course of SCIT. The graphs show the symptom scores before and after SCIT - in SCIT-treated patients without neosensitization to treatment-related components (grey graphs; n = 25) and in SCIT-treated patients with *de novo* sensitization to therapy-related components (black graphs, n = 7). Only in SCIT-treated patients without neosensitization to treatment-related components, a significant reduction of symptom severity is observed after SCIT (*p < 0.05).

In order to assess the clinical outcome of SCIT, the clinical symptoms were assessed before and after SCIT by a standardized questionnaire. 25 patients without new sensitizations against the treated allergen source had completed the questionnaire before and after SCIT: As shown in Fig. 4, a strong and statistically significant reduction in severity was found in all symptoms (p < 0.05 in all symptoms). Among the 12 patients with treatment-related neosensitization, only seven completed the questionnaire before and after treatment. These seven patients reported on a smaller relief of symptoms, compared to SCIT-patients without neosensitization. However, these data have to be interpreted with precaution due to the very small sample size. Comparing the symptom scores at the initial consultation, it becomes obvious that patients with subsequent neosensitization to SCIT-related allergen components had less severe symptoms than those without. However, this difference is statistically not significant.

### Non-therapy-related neosensitization throughout therapy

Besides the sensitization patterns against allergen components, which the treatment was focused on, we also analysed the sIgE against >150 allergen sources other than *Fagales* or *Poaceae* or HDM. In order to avoid misinterpretation of cross-reactions, we did not count new sIgE towards allergen components of which homologues had already been recognized beforehand at the initial measurements. We considered new sIgE only as sign of neosensitization, if they were directed towards allergen components, which are genuine for an allergen source, which was not recognized by sIgE in the first serum sample.

In the control group, we found neosensitizations to new allergen sources (other than *Fagales* or *Poaceae* or HDM) in one single adult patient (affecting ash, Fra e 1). However, in the SCIT-treated group, new sIgE to allergen sources other than the treated were seen in several cases: Nine adults and four children were affected and newly sensitized against olive, dog, bee, wesp and cow (adults) and cat, cow, carp and ragweed (children). Five of the 13 SCIT-treated patients with neosensitization to untreated allergens were also affected by therapy-related neosensitization (four adults and one child).

## Discussion

Allergen SIT is the only disease-modifying treatment of IgE-mediated allergies. However, despite the enormous clinical benefit achieved by SIT, the treatment-related risks should not be underestimated. In the context of SIT, the mostly discussed and cited risk surely is anaphylaxis due to the administration of high allergen doses. However, the present study brings up another potential risk, the induction of new sensitizations to formerly unrecognized allergen components contained in the vaccine. Although there are increasing attempts to standardize test and treatment allergens, it is not possible at present to compare different vaccines as manufacturers use different systems to measure allergen concentrations^19^. As vaccines are prepared from natural allergen sources, they contain a large number of various allergen components at unknown doses^20,21^, which might induce new sensitization, especially as administered together with adjuvants.

In our birch-allergic study population, the prevalence of sIgE against different birch components was comparable with results from other studies on patients with allergy against *Fagales*^22^. Further, the prevalence of sIgE in our patients with allergy to grass^23^ or HDM^24^ was also in line with the literature. However, in the course of SCIT, the pattern of sIgE against components of the treated allergen source changed. In our 51 SCIT-treated patients, we found neosensitization to treatment-related allergen components in 24%. As the development of IgE antibodies against new allergen components during immunotherapy might be age-dependent^25^, we performed a subgroup-analysis of all patients older than 11 years, which resulted in a similar rate of therapy-related neosensitization among adults at 20%. In the control group without SCIT-treatment, neosensitization was limited to two cases – both children. The fact that no adult of the control group was affected by *de novo* sensitization – even despite a longer observation period – is in line with the literature: Without SCIT, *de novo* sensitizations rarely occur in allergic adults, possibly due to the maturity of the adult immune systems and the rather low concentrations of allergens by natural exposure^26^. For HDM, this is confirmed by the recent results from a large cohort study by Posa *et al*.^24^. The evolution of IgE responses to different HDM allergens increased throughout the first decade of life. Thereafter, hardly any new HDM-related sensitization occurs.

In the SCIT group, the neosensitization to treatment-related major allergen components was predominantly accompanied by strong increases of corresponding sIgG. In contrast, this induction of sIgG was almost missing in the two untreated children, who experienced neosensitization to HDM major allergen components although they had not undergone SCIT. This discrepancy between the two groups confirms our assumption that neosensitization in the SCIT group might be treatment-induced whereas neosensitization found in the two young control patients might be due to the natural allergic march in children.

Consequently, we have reasons to believe that the rate of 20% of neosensitization to HDM allergens in our SCIT-treated adult study population might be treatment-induced. A clear clinical predictor for an increased risk of therapy-induced neosensitization could not be identified.

However, one has to keep in mind that – despite great effort to match study group and control cohort – different phenotypes might be present in both groups: Although all patients had clinically relevant sensitizations, control patients decided against a SCIT, for different reasons, which could not be identified in most cases due to the retrospective study design. In some cases, contraindications such as insufficiently controlled asthma might have precluded a SCIT. Other control patients, however, might have had weaker allergic symptoms. Therefore, it cannot be excluded that patients in the SCIT-group might be of a different phenotype more susceptible to acquire new sIgE sensitizations induced by SCIT. On the other hand, the comparison of all available demographic, clinical and serological characteristics of SCIT-groups und control cohorts led to very similar results. Nevertheless, this limitation by selection bias could only be overcome by a prospective randomized study design.

Compared to the wide application of SCIT, the number of studies dealing with a potential therapy-induced neosensitization is small, probably due to the difficulty to recruit patients, especially for the control group. Even less data, however, are found concerning the clinical relevance of SCIT-caused neosensitization. Several studies could demonstrate that the complexity of sIgE responses to HDM allergens is associated with clinical outcomes^24,27^. Children with asthma show expanded sIgE repertoires with respect to the number of recognized allergen components^28^. This observation is based on data from patients, who did not undergo SCIT and whose sIgE derive, therefore, from sensitization to the natural allergen source. This is the reason why these results cannot be transferred one-to-one to patients with therapy-induced neosensitization. However, this concordance of complex sIgE repertoires and clinical outcome should be investigated in cases of therapy-induced expansion of sIgE pattern.

In order to detect a potential clinical impact of SCIT-induced neosensitization, we compared the change in symptom scores throughout SCIT between patients with therapy-related neosensitization and patients without therapy-related neosensitization. Interestingly, patients without therapy-induced neosensitization reported a stronger (and statistically significant) improvement of all symptoms compared to patients with treatment-related neosensitization, who claimed less relief from symptoms despite SCIT. At first sight, this striking result appears hardly explainable: As soon as treatment induces new sIgE reactivity, one might expect the production of blocking sIgG-subclasses leading to a tolerance, which neutralizes the newly acquired sensitization. However, it is well known that allergen extracts contain very various concentrations of different allergen components^21^. In solutions for SCIT, the concentration of major allergens is measured by all manufacturers and validated as high enough to induce blocking sIgG. However, concerning intermediate and minor allergens, manufacturers do not standardize their treatment solutions. Therefore, it cannot be excluded that low concentrations of intermediate or minor allergens induce sIgE sensitization, but are too low for the production of blocking sIgG. Indeed, our data confirm that the majority of patients treated by SCIT showed sIgG to major allergen components after treatment. To the allergen components Der p 7, 10 and 11, however, sIgG were not seen in any case despite treatment by SCIT and even despite newly induced sIgE to these allergen components. Concerning minor allergens components of Fagales and Poaceae, a small fraction of patients developed sIgG. As even intermediate and minor allergens, as Der p 5, seem to be associated with strong allergic reactions^29^, this lack of sIgG despite newly induced sIgE might explain why patients with neosensitization during SCIT might have a worse clinical outcome compared to SCIT-treated patients without neosensitization. Nevertheless, our sample size is too small to draw firm conclusions, but still gives reason to investigate the clinical course of patients with therapy-induced neosensitizations in a larger study population.

Regarding SCIT-related *de novo* sensitizations to new allergen sources, conflicting results are found in the literature: Whereas older studies and consensus papers suggest that SIT has preventive effects on the onset of new allergies^30–32^, Tella *et al*. found at least the same rate of new sensitization during three to five years of SIT compared to allergic patients without SCIT over the same period of time^33^. Besides the well-known publication bias with positive results being more likely to be published than negative results, a comprehensive review on this aspect of SIT found only low strength evidence for the preventive aspect of SIT on the development of new allergen sensitization^34^.

In our study population, we found new sensitizations to other allergens than the treated primarily in the SCIT-treated group (25% vs. 3% among controls, p = 0.007). These *de novo* sensitizations also applied to widespread allergen sources. With regard to the affected allergens – especially olive and dog – it is rather unlikely that adults were not exposed to these allergens beforehand. Further, it remains remarkable that such phenomenon of “late first exposures” was significantly less present in the control group. On the one hand, one could argue that this result might be a hint that our SCIT-treated group was, in general und independently from therapy, more susceptible to new sensitizations than the controls. On the other hand, the comparison of clinical and serological characteristics of both patient cohorts at the first consultation revealed very similar results. In consequence, this good comparability of both cohorts justifies raising concerns, whether SCIT might make patients even more susceptible to new therapy-unrelated allergen sensitizations as already assumed by other studies^16,35^.

## Conclusion

During SCIT with *Fagales* or *Poaceae* or HDM, neosensitization to components of the treated allergen source was found in 20% among adults, whereas, in the control group, no adult patient was affected by *de novo* sensitization to *Fagales* or *Poaceae* or HDM. Although the present study is retrospective and the sample size not huge (n = 51 and n = 33, respectively), the discrepancy is too high to be neglected and emphasizes the conduction of prospective randomised studies on the risk of neosensitization during SCIT compared to symptomatic treatment.

In case of SCIT-provoked neosensitization, clinical relevance directly during SCIT or shortly thereafter is not excluded, especially in cases of *de novo* sensitization to intermediate or minor allergens of which the concentration in the treatment solutions of our study was apparently high enough to induce sIgE, but too low for the induction of blocking sIgG. Even in cases of neosensitization to major allergens and a corresponding induction of sIgG, clinical relevance appears imaginable a decade after SCIT, when treatment effects usually decrease due to fading neutralizing sIgG.

As no recombinant allergen components are available for *in vivo* testing, clinical observational studies across the end of SCIT are necessary to estimate the clinical relevance of potential therapy-induced neosensitization. SIT remains the undisputed gold-standard therapy of IgE-mediated allergies; however, the results of the present study reinforce the high significance of recent attempts towards personalized recombinant vaccines.

## Conflicts of Financial and Non-Financial Interest

D.G. has received speaker honoraria from ALK-Abelló and Bencard Allergy and financial support for attending symposia from HAL Allergy, Phadia diagnostics and Shire. C.H. has received financial support for attending symposia from HAL Allergy, Shire and Lofarma. S.B. has received speaker honoraria from ALK-Abelló, HAL Allergy, Allergopharma, Bencard Allergy and Phadia diagnostics and is member of the advisory board of Bencard Allergy. M.G. has received speaker honoraria and financial support for attending symposia from ALK-Abelló, Allergopharma, Bencard Allergy, HAL Allergy, Phadia diagnostics, Shire and Stallergenes and is member of the advisory board of ALK-Abelló. However, all these potentially conflicting interests did not have any influence on the study. There were no further financial or non-financial competing interests. No funding of the study.
